# Malignant testicular unclassified sex cord stromal tumor: a case report

**DOI:** 10.1186/s13256-022-03640-z

**Published:** 2022-11-06

**Authors:** Shingo Morinaga, Shigeyuki Aoki, Toyonori Tsuzuki, Keiko Kanematsu, Naoki Kawai, Sayuri Sato, Kotomi Nakamizu, Kana Goto, Miho Niwa, Ikuko Nomura, Tomoko Sawada, Ruriko Futamachi, Fujio Morita, Yoshiaki Yamada

**Affiliations:** 1 The Department of Urology, Japan Community Health Care Organization, Kani Tono Hospital, 1221-5 Dota, Kani, Gifu 509-0206 Japan; 2 The Department of Surgical Pathology, Aichi Medical University Hospital, 1-1 Yazakokarimata, Nagakute, Aichi 480-1195 Japan; 3 The Department of Clinical Laboratory, Japan Community Health Care Organization, Kani Tono Hospital, 1221-5 Dota, Kani, Gifu 509-0206 Japan; 4 The Division of Nursing, Japan Community Health Care Organization, Kani Tono Hospital, 1221-5 Dota, Kani, Gifu 509-0206 Japan; 5 The Division of Hospital and Clinic Coordination, Japan Community Health Care Organization, Kani Tono Hospital, 1221-5 Dota, Kani, Gifu 509-0206 Japan; 6 The Division of Radiology, Japan Community Health Care Organization, Kani Tono Hospital, 1221-5 Dota, Kani, Gifu 509-0206 Japan

**Keywords:** Unclassified sex cord stromal tumor, Testicular tumor

## Abstract

**Background:**

Most testicular tumors are germ cell tumors; sex cord stromal tumors are infrequent, accounting for only 3–5% of testicular tumors. Unclassified sex cord stromal tumors are extremely rare. Generally, 10% of sex cord stromal tumors are malignant. We report a case of malignant unclassified sex cord stromal tumor with retroperitoneal lymph node metastasis at first visit and a corresponding literature review.

**Case presentation:**

A 72-year-old Japanese man visited our department primarily for indolent right scrotum enlargement in September 2020. Blood biochemistry examination, urinalysis, and tumor markers (alpha-fetoprotein, human chorionic gonadotropin, and lactate dehydrogenase) showed no abnormal findings. Contrast-enhanced computed tomography showed enlarged para-aortic lymph node (18 × 16 and 10 × 10^2^ mm); a 50 × 45^2^ mm mass with uneven contents was found in the right testicle. The patient underwent inguinal orchiectomy in September 2020. As per immunohistochemistry, the tumor cells were diffusely positive for SF-1 and Ki-67, partially positive for inhibin, and negative for CAM5.2, CK7, CK20, C-KIT, CD30, LCA, GATA-3, TTF-1, and PAX8. Calretinin was expressed in approximately 5% of tumor cells; thus, sex cord/gonadal stroma components were considered to be involved. The final pathological diagnosis was unclassified malignant sex cord stromal tumor. The patient was diagnosed with pT1, N1, M0, S0, and tumor–node–metastasis stage IIA disease. The patient received postoperative chemotherapy with four courses of etoposide and cisplatin therapy from November 2020. Post-chemotherapeutic computed tomography showed new metastatic lesions including lung, liver, pancreas, and para-aortic lymphadenopathy, which increased in size. Disease progression was observed. Cancer genome research was performed using the OncoGuide National Cancer Center oncopanel system; however, no gene mutation for which the drug could be expected to be effective was found. The patient opted for best supportive care at a nearby hospital and died from cancer progression in January 2022.

**Conclusion:**

We encountered a case of malignant testicular unclassified sex cord stromal tumor pathologically diagnosed as testicular tumor with retroperitoneal lymph node metastasis in a patient who underwent inguinal orchiectomy. Future data collection is necessary to establish multimodality therapy for malignant testicular unclassified sex cord stromal tumor.

## Background

The World Health Organization (WHO) classification is widely used for pathological classification of testicular tumors [1], which are mainly divided into germ cell tumors and SCSTs. Most testicular tumors are germ cell tumors whereas SCSTs are infrequent, accounting for only 3–5% of testicular tumors [2]. Approximately 10% of SCSTs are malignant [3].

Leydig cell tumors are the most common SCSTs, followed by Sertoli cell tumors, mixed type consisting of multiple tissue components, and unclassified. Reports of unclassified SCSTs are rare.

We report the case of a patient who underwent inguinal orchiectomy at first visit for a testicular tumor with RPLN metastasis and was diagnosed with unclassified SCST by pathological diagnosis; we also report a corresponding literature review.

## Case presentation

A 72-year-old Japanese man visited our department primarily for indolent right scrotum enlargement in September 2020. The patient had undergone appendectomy and left inguinal hernia surgery 50 and 10 years ago, respectively. The patient has been receiving oral treatment for benign prostatic hyperplasia, diabetes, and hypertension for 5 years.

At first visit, the patient’s height and weight was 163 cm and 50 kg, respectively. His blood pressure was 136/80 mmHg, pulse was regular at 62 beats/min, and body temperature was 36.3 °C.

He had no history of smoking or drinking, and there was nothing significant in his family history.

Physical examination revealed that his right testicle was elastic, hard, and enlarged to a size of 50 × 50 mm^3^. No obvious lymphadenopathy on the body surface was palpable, and no gynecomastia was found. Neurological examination revealed no abnormalities. Blood biochemistry, urinalysis, and tumor marker levels (AFP, HCG, and LDH) showed no abnormal findings.

Ultrasound findings showed a mosaic shadow inside the right testicle with no abnormal findings in the left testicle.

**Fig. 1:**
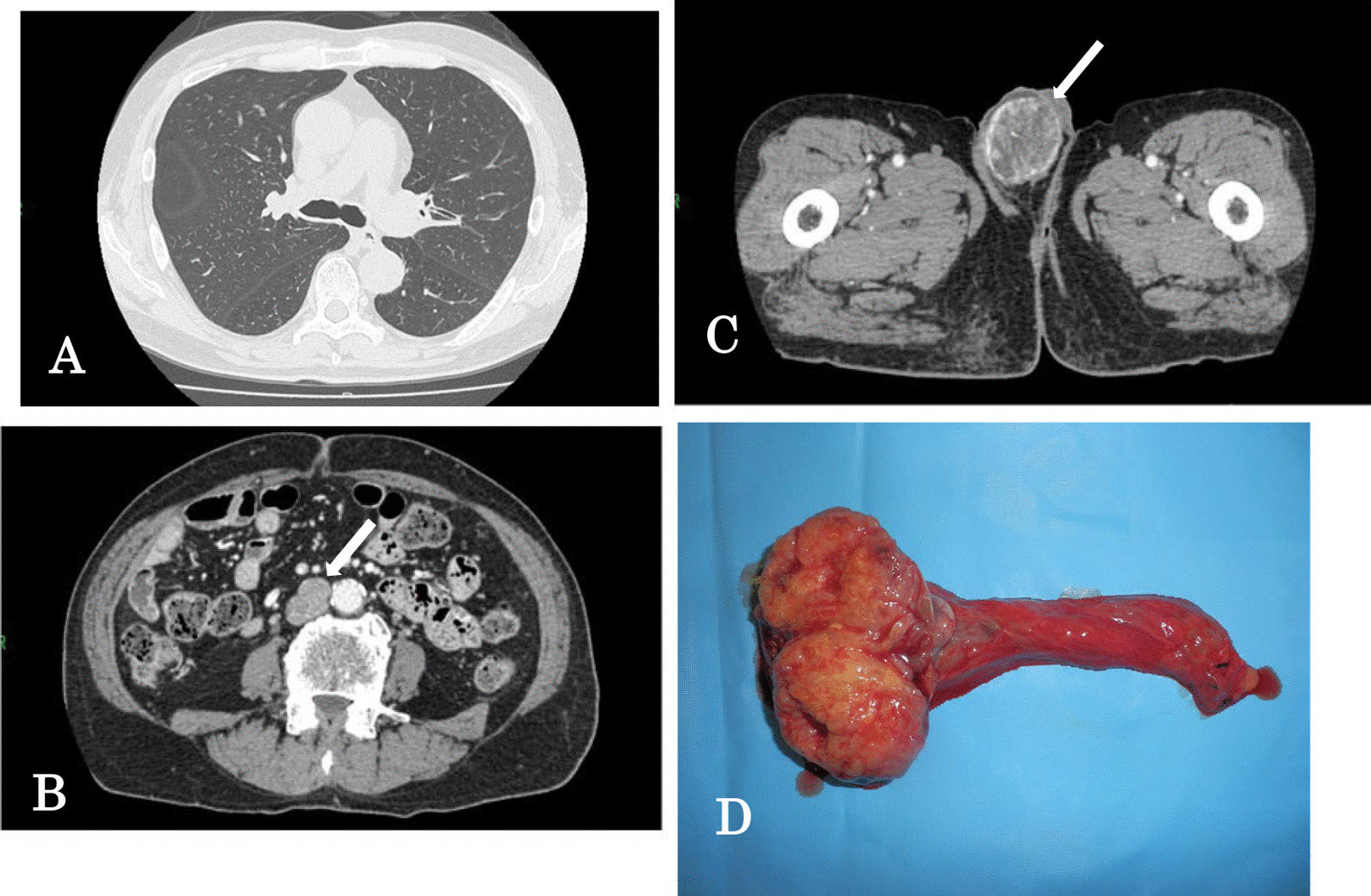
Lung CT showed no evidence of any coin lesions (**A**). Contrast-enhanced CT showed enlargements of the para-aortic lymph node (**B**, 18 × 16 mm^2^, white arrow), and a 50 × 45 mm^3^ mass with uneven contents was found in the right testicle (**C**, white arrow). The diagnosis was a testicular tumor (cT1, N1, M0, S0, and TNM stage IIA). The resected testicular tumor was 55 × 45 × 40 mm^3^ in size and 63 g in weight. The cut surface was yellowish-white with bleeding and solid, and the testicular tumor was localized in the testicle (**D**)

Lung CT showed no evidence of coin lesions or effusion (Fig. 1A). Contrast-enhanced CT showed an enlarged para-aortic lymph node (18 × 16 and 10 × 10 mm^2^) (Fig. 1B), and a 50 × 45 mm^3^ mass with uneven contents was found in the right testicle (Fig. 1C). No evidence of distant metastasis was found during the other diagnostic procedures.

For the right testicular tumor, the TNM classification was cT1, N1, M0, S0, and the clinical stage was IIA [1]. The patient underwent inguinal orchiectomy in September 2020. The resected testicular tumor was 55 × 45 × 40 mm^3^ in size and 63 g in weight. The cut surface was yellowish-white with bleeding and solid, and the testicular tumor was localized in the testicle (Fig. 1D).

Histopathological findings showed a solid tumor confined to the testis, with distinct nucleoli and large nuclei, proliferating in a nest-like manner with some necrosis, and no specific differentiation tendency was observed.

**Fig. 2:**
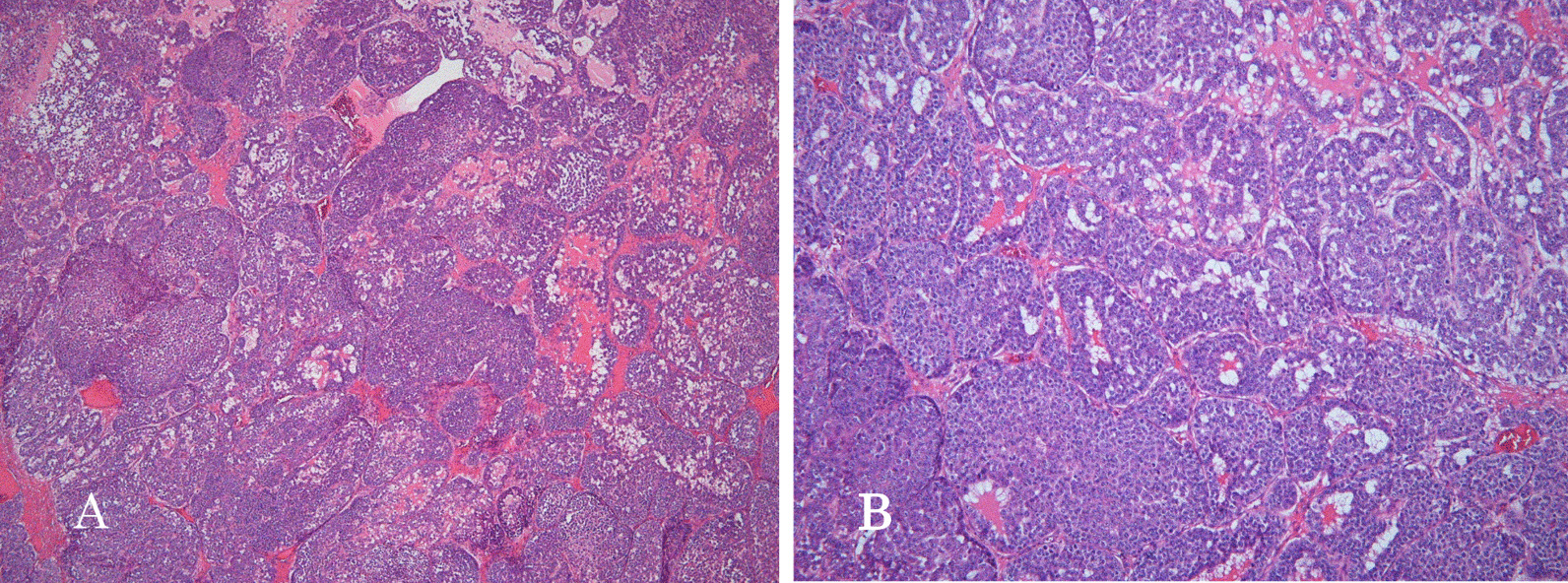
Histopathological findings showed a solid tumor confined to the testis; the tumor cells have distinct nucleoli and large nuclei; they proliferate in a nest-like manner with some necrosis, and no specific differentiation tendency was observed. Vascular infiltration of tumor cells was observed following hematoxylin and eosin staining (**A**: × 40, **B**: × 100)

Vascular infiltration of tumor cells was also observed. The tumor was poorly differentiated (Fig. 2A, B).

**Fig. 3:**
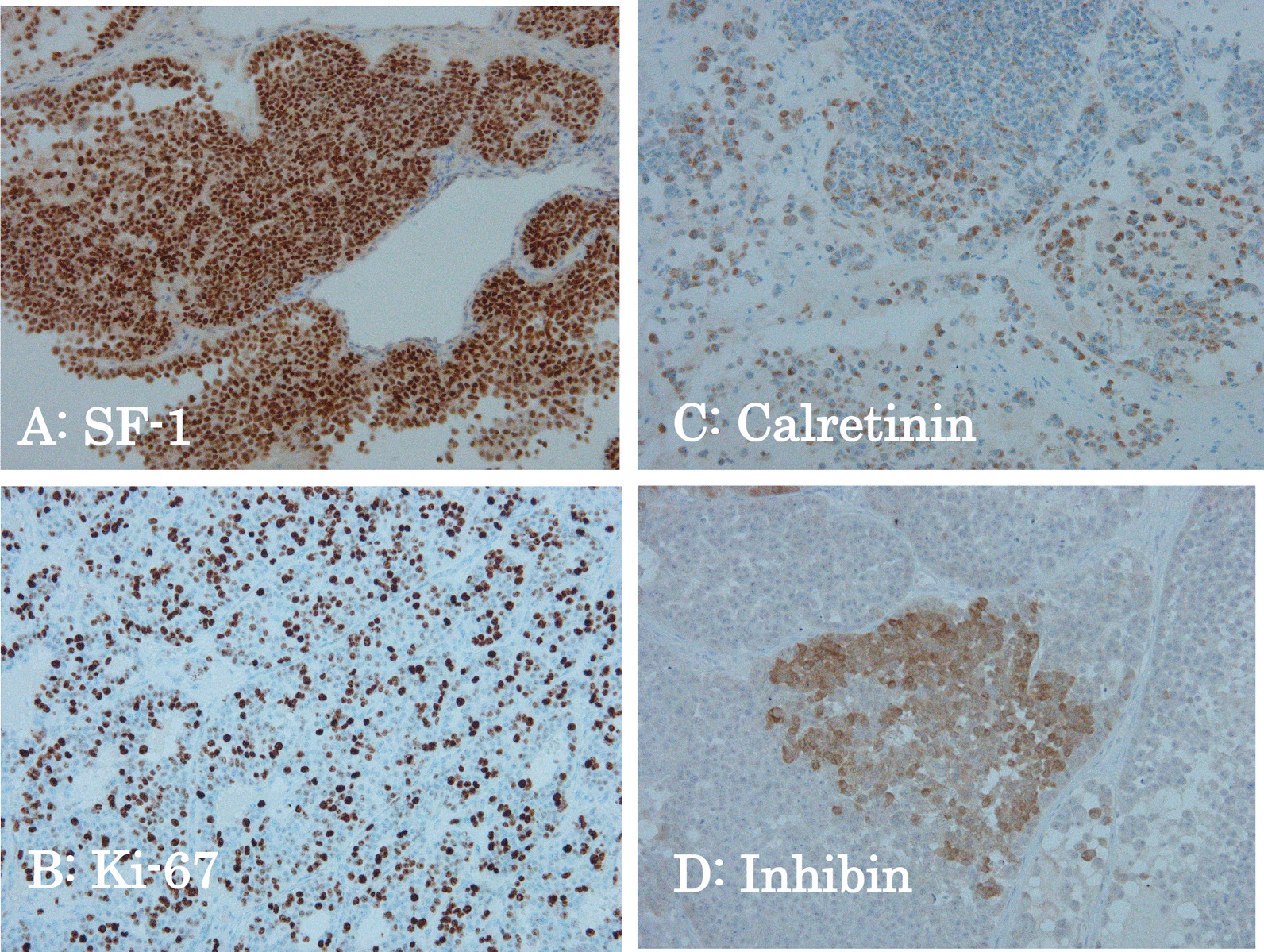
As per immunohistochemistry, the tumor cells were diffusely positive for SF-1 (**A**, × 200) and Ki-67 (**B**, × 200), and partially positive for inhibin (**C**, × 200), and calretinin was expressed in about 5% of tumor cells (**D**, × 200)

Immunohistochemistry showed that the tumor cells were diffusely positive for SF-1 (Fig. 3A) and Ki-67 (Fig. 3B), partially positive for inhibin (Fig. 3C), and negative for CAM5.2, CK7, CK20, C-KIT, CD30, LCA, GATA-3, TTF-1, and PAX8. Calretinin was expressed in approximately 5% of tumor cells (Fig. 3D), thus sex cord/gonadal stroma components were considered to be involved.

The final pathological diagnosis was malignant unclassified SCST.

The tumor did not extend into the tunical albuginea, and vascular infiltration was found in one part of the tumor. The spermatic cord stumps were negative. Thus, the tumor was diagnosed as pT1, N1, M0, S0, and TNM stage IIA.

The patient received postoperative chemotherapy with four courses of etoposide and cisplatin therapy from November 2020.

**Fig. 4:**
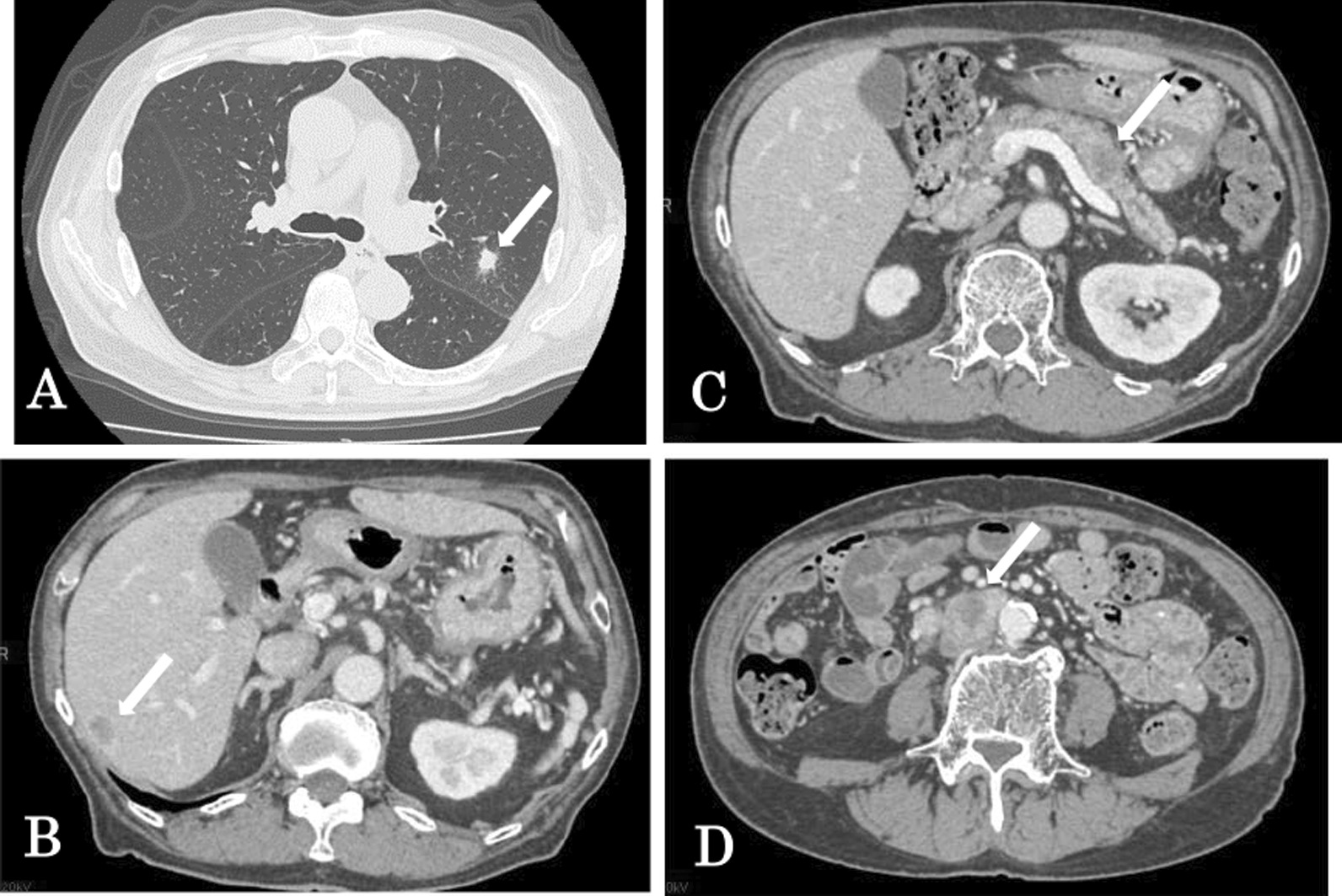
Contrast-enhanced CT after four courses of etoposide and cisplatin chemotherapy showed new metastatic lesions in the lung (**A**, 14 × 10 mm^2^, white arrow), liver (**B**, 16 × 12 mm^2^, white arrow), and pancreas (**C**, 18 × 15 mm^2^, white arrow), and increased para-aortic lymphadenopathy was also observed (**D**, 31 × 26 mm^2^, white arrow)

Post-chemotherapeutic CT showed new metastatic lesions in the lung (14 × 10 mm^2^) (Fig. 4A), liver (16 × 12 mm^2^) (Fig. 4B), and pancreas (18 × 15 mm^2^) (Fig. 4C), and para-aortic lymphadenopathy was increased (31 × 26 mm^2^) (Fig. 4D). Disease progression was observed.

Subsequently, cancer genome research was performed using the OncoGuide NCC oncopanel system, but no gene mutation for which the drug could be expected to be effective was found.

Additionally, paclitaxel, ifosfamide, and cisplatin therapy was proposed as second-line chemotherapy, but the patient refused it and was transferred to a nearby hospital in August 2021 for best supportive care. However, in January 2022, the patient died of cancer progression at a transfer hospital.

## Discussion

The WHO classification is widely used for pathological classification of testicular tumor [1]. Testicular tumors are mainly divided into germ cell tumors and SCSTs. Most testicular tumors are germ cell tumors while SCSTs are infrequent, accounting for only 3–5% of testicular tumors [2]. Generally, 10% of SCSTs are malignant [3].

Inguinal orchiectomy is indicated in SCST, and definitive diagnosis is made by pathological examination. However, in case of Leydig cell tumor and Sertoli cell tumor, it is difficult to distinguish benign and malignant tumors using pathological diagnosis, and there are many cases of diagnosis based on the presence of metastasis at follow-up.

Kim *et al.* [4] defined malignancy criteria as tumor diameter of 5 cm, necrosis, lymphatic and/or vascular invasion, nuclear atypia, and high mitotic index. Additionally, Cheville *et al.* [5] demonstrated that the proliferation rate (determined immunohistochemically with Mindbomb homolog-1) and DNA ploidy (as evaluated by static image analysis) are additional descriptive factors for malignancy.

Metastatic disease is present in 20% of patients with SCSTs at initial diagnosis. In the remaining 40%, metastatic disease develops within the first 2 years. Metastatic disease frequently involves the lymph nodes (70%), particularly the retroperitoneal and inguinal lymph nodes. Other frequent metastatic regions are the liver (45%), lung (40%), and bones (25%) [1, 6]. In the present case, RPLN metastasis was observed at the first visit itself.

Some investigators have reported that metastatic SCST progresses rapidly and has poor prognosis. They also emphasized that RPLN dissection, adjuvant chemotherapy, and systemic chemotherapy and radiotherapy, that is, all treatment alternatives after orchiectomy, make an insignificant contribution to disease prognosis [3, 7, 8]. Silberstein *et al.* described that, considering the lack of effective alternative treatments, early RPLN dissection may be beneficial in clinical stage IIA disease [9]. However, evidence supporting RPLN dissection for this rare tumor is lacking.

Herein, four courses of etoposide and cisplatin therapy were administered on the basis of a report that chemotherapy was effective for SCST [10–12]; however, lung, liver, and pancreatic metastases appeared, and the RPLN metastasis site also increased in size. Disease progression was observed.

It has been reported that immunosuppression associated with the use of nonsensitive anticancer drugs may worsen the prognosis of malignant tumors including those of the genitals, and administration should be investigated in the future [13].

Recently, with the progress in cancer genome research, it has become possible to individually select effective cancer therapeutic agents. In this case, cancer genome research using the OncoGuide NCC oncopanel system [14] was performed, but no gene mutation for which the drug could be expected to be effective was found.

For metastatic SCSTs, it is necessary to establish an effective treatment strategy immediately. However, the rarity of these tumors, absence of prospective studies, and lack of randomized studies with adequate patient numbers have resulted in incorrect implementation of various treatments, causing inaccurate diagnosis and preventing the establishment of a valid treatment approach.

Only one case of unclassified malignant SCST has been reported in Japan, and further data collection is necessary to establish the most effective treatment.

## Conclusion

We report a case of malignant testicular unclassified SCST through pathological diagnosis of a testicular tumor with RPLN metastasis in a patient who underwent inguinal orchiectomy.

Further data collection is necessary to establish a multimodality therapy for malignant testicular unclassified SCST.

## Data Availability

Not applicable.

## Abbreviations

SCST: Sex cord stromal tumor
RPLN: Retroperitoneal lymph node
AFP: Alpha-fetoprotein
HCG: Human chorionic gonadotropin
LDH: Lactate dehydrogenase
CT: Computed tomography
WHO: World Health Organization
NCC: National Cancer Center
